# Supplementary material for: Guiding Drug Repositioning for Cancers Based on Drug Similarity Networks
Source: Int J Mol Sci. 2023 Jan 23;24(3):2244. doi: 10.3390/ijms24032244 (PMC9917231; doi:10.3390/ijms24032244)
Supplement: Supplementary file 1 [file ijms-24-02244-s001.zip › ijms-2091704-supplementary.pdf]

Guiding drug repositioning for cancers based on drug similarity networks

Shimei Qin [1,†], Wan Li [1,†], Hongzheng Yu [1], Manyi Xu [1], Chao Li [1], Lei Fu [1], Shibin Sun [1], Yuehan He [1], Junjie Lv [1], Weiming He [2], and Lina Chen [1,*]

Supplementary Materials

Table S1

Known therapeutic drugs for NSCLC.

Accession Number	Drug name
DB00112	Bevacizumab
DB00317	Gefitinib
DB00361	Vinorelbine
DB00441	Gemcitabine
DB00530	Erlotinib
DB00642	Pemetrexed
DB01229	Paclitaxel
DB01248	Docetaxel
DB08865	Crizotinib
DB08916	Afatinib
DB09035	Nivolumab
DB09037	Pembrolizumab
DB09063	Ceritinib
DB09079	Nintedanib
DB09330	Osimertinib
DB09559	Necitumumab
DB11363	Alectinib
DB11714	Durvalumab
DB11791	Capmatinib
DB11963	Dacomitinib
DB12130	Lorlatinib
DB12267	Brigatinib
DB15685	Selpercatinib
DB11595	Atezolizumab

Table S2

Potential repositionable drugs for colorectal cancer.

Average Rank	Accession Number	Drug Name	Evidence
3	DB09559	Necitumumab	PMID: 21154125,PMID: 26766738
12	DB08870	Brentuximab vedotin	PMID: 30993587
12	DB12498	Mogamulizumab	PMID:31801624,PMID: 34916725,PMID: 31455681
29	DB09330	Osimertinib	PMID: 31409796
29	DB11737	Icotinib	PMID: 25572529,PMID: 35250582
29	DB11828	Neratinib	PMID: 26243863,PMID: 30203445
29	DB11963	Dacomitinib	PMID: 27733479,PMID: 22249430
29	DB13164	Olmutinib	Unconfirmed
30	DB01005	Hydroxyurea	Unconfirmed

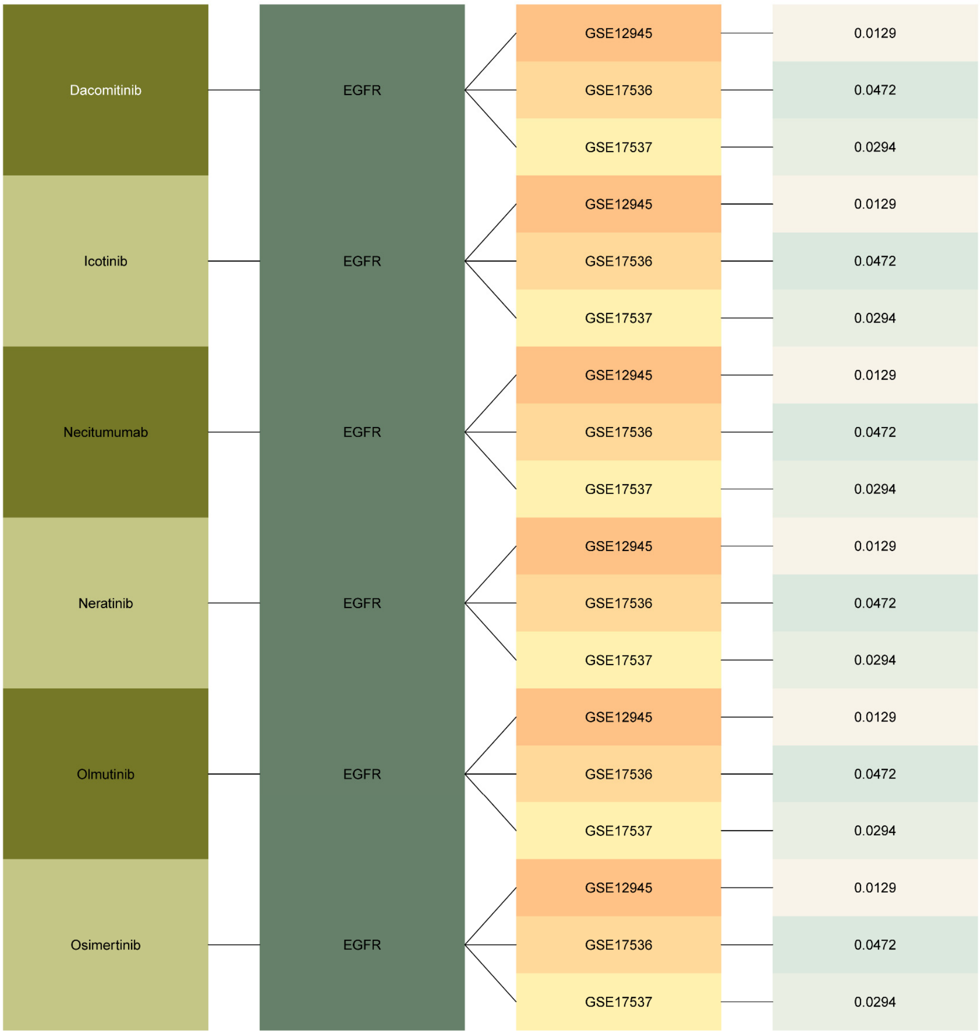

Figure S1

Prognostic analysis of potential repurposable drugs in colorectal cancer.

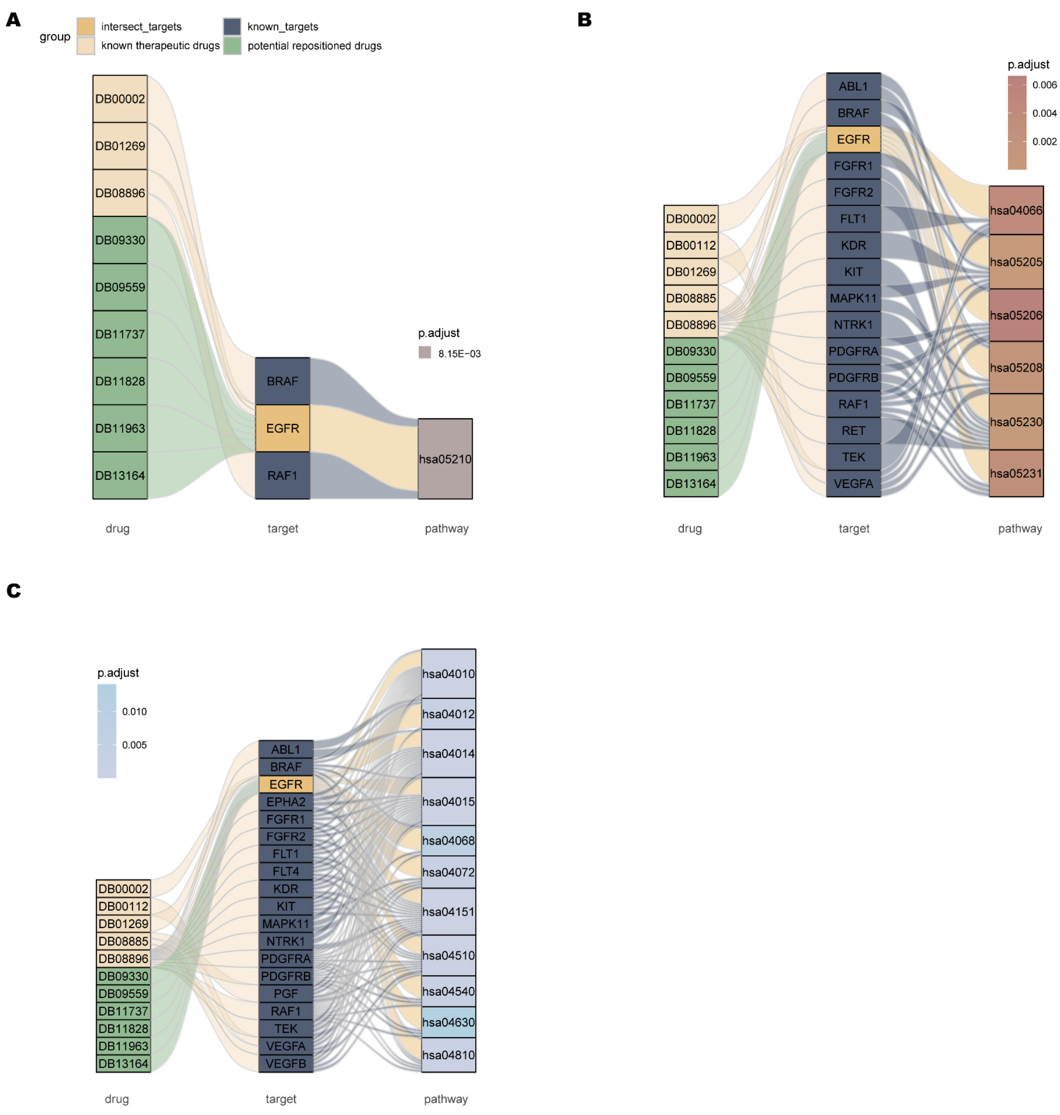

Figure S2

KEGG enrichment analysis of potential repositionable drugs for colorectal cancer. **(A)** colorectal cancer pathway. **(B)** Cancer progression-related pathway. **(C)** Cell process-related pathway.

Document S1

The whole set of findings on colorectal cancer.

1. Results

1.1. Stable drug candidates in unweighted pattern

For colorectal cancer, 11 known therapeutic drugs from the DrugBank database were selected as a seed set (Table S3). The leave-one-out cross validation was performed to calculate the recall rate of 11 known therapeutic drugs of colorectal cancer. 9 of these drugs were recalled in corresponding identified top 5% of drugs, with a recall rate of 0.82 (Figure S3A).

The 211 drugs (top 5%) were screened when all known therapeutic drugs were used as seeds. The delete-n-out strategy stopped after removing 6 seeds. 206-211 and 201-311 drugs were captured as top 5% by the delete-1-out and delete-6-out strategies, respectively (Figure S3B). Eleven and 454 were the frequency thresholds for the delete-1-out and delete-6-out strategies, respectively (Figure S3C). The 182 and 35 drug candidates were captured corresponding to the delete-1-out and delete-6-out strategies. The final 35 drug candidates were defined as the stable drug candidates screened in the unweighting pattern. Each of the 35 stable drug candidates had an average ranking in the top 50 (Figure S3D).

Table S3

Known therapeutic drugs for colorectal cancer.

Accession number	Drug name
DB00002	Cetuximab
DB00112	Bevacizumab
DB00293	Raltitrexed
DB00432	Trifluridine
DB00762	Irinotecan
DB01269	Panitumumab
DB06186	Ipilimumab
DB08885	Aflibercept
DB08896	Regorafenib
DB09035	Nivolumab
DB09256	Tegafur

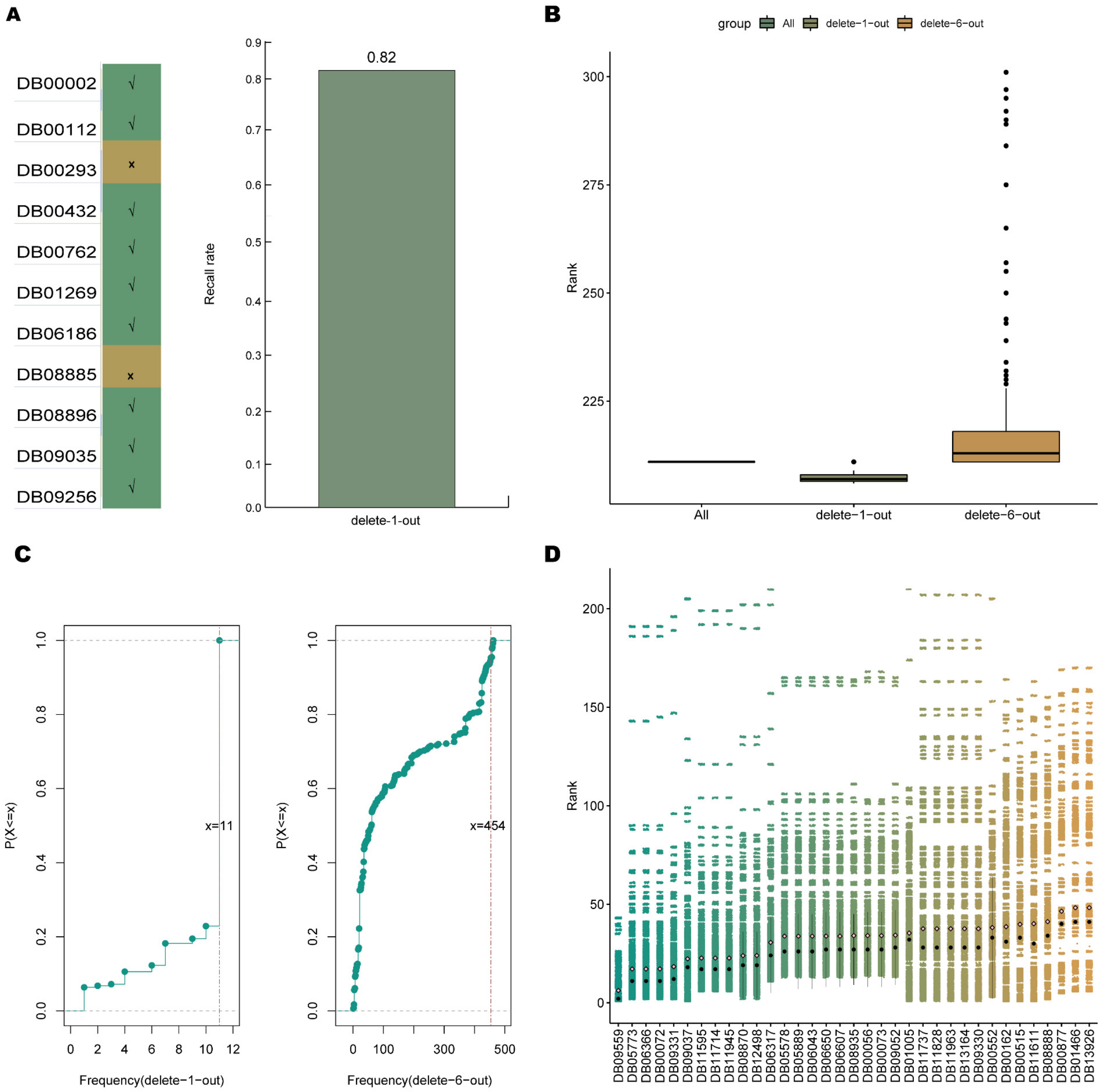

Figure S3

Stable drug candidates in unweighted pattern. **(A)** The recall of the leave-one-out cross validation. "√" means the drug was recalled in the top 5% of drugs, and "×" denotes that the drug was not recalled. **(B)** Distribution of the number of the top 5% of drugs. "ALL" represents all known therapeutic drugs as a seed set. **(C)** Frequency distribution. The orange dashed line shows the 95th percentile of the distribution. **(D)** Distribution of the ranking for the stable drug candidates in the unweighted pattern. Where the average ranking is marked by a pink diamond and the median is marked with a black dot. In addition, the Venn diagram of the top 5% of drugs filtered with different number of seeds removed is inserted.

1.2. Stable drug candidates in weighted pattern

The recalls of the leave-one-out cross validation for all weighted patterns were 0.91 (for the 29% weighting patterns) or 0.82 (for the 71% weighting patterns) (Figure S4A). 205-229 drugs (top 5%) were recognized through 54 weighting patterns, when all known therapeutic drugs were taken as the seed set (Figure S4B).

The delete-n-out strategy was implemented in each weighting pattern with a cutoff condition of deleting 3 seeds. 206-296 drugs were recognized as top 5% by delete-1-out strategy in different weighting patterns, 208 to 298 drugs were detected as top 5% through delete-3-out (Figure S4B). The delete-1-out has a frequency threshold of 11 for all 54 weights (Figure S4C). The frequency thresholds ranged from 161-165 for delete-3-out in different weighting patterns (Figure S4D). 121-204 and 25-177 drugs were identified by implementing the delete-1-out and delete-3-out strategies in 54 weighting patterns, respectively (Figure S4E-F). The drugs as the minimum set identified by performing the delete-3-out strategy in each weighting pattern were defined as candidates for that pattern. The final 16 candidates that served as the intersection of the candidates captured by all weighting patterns were considered as stable drug candidates screened in the weighting pattern (Figure S4G). All stable drug candidates were ranked in the top 50.

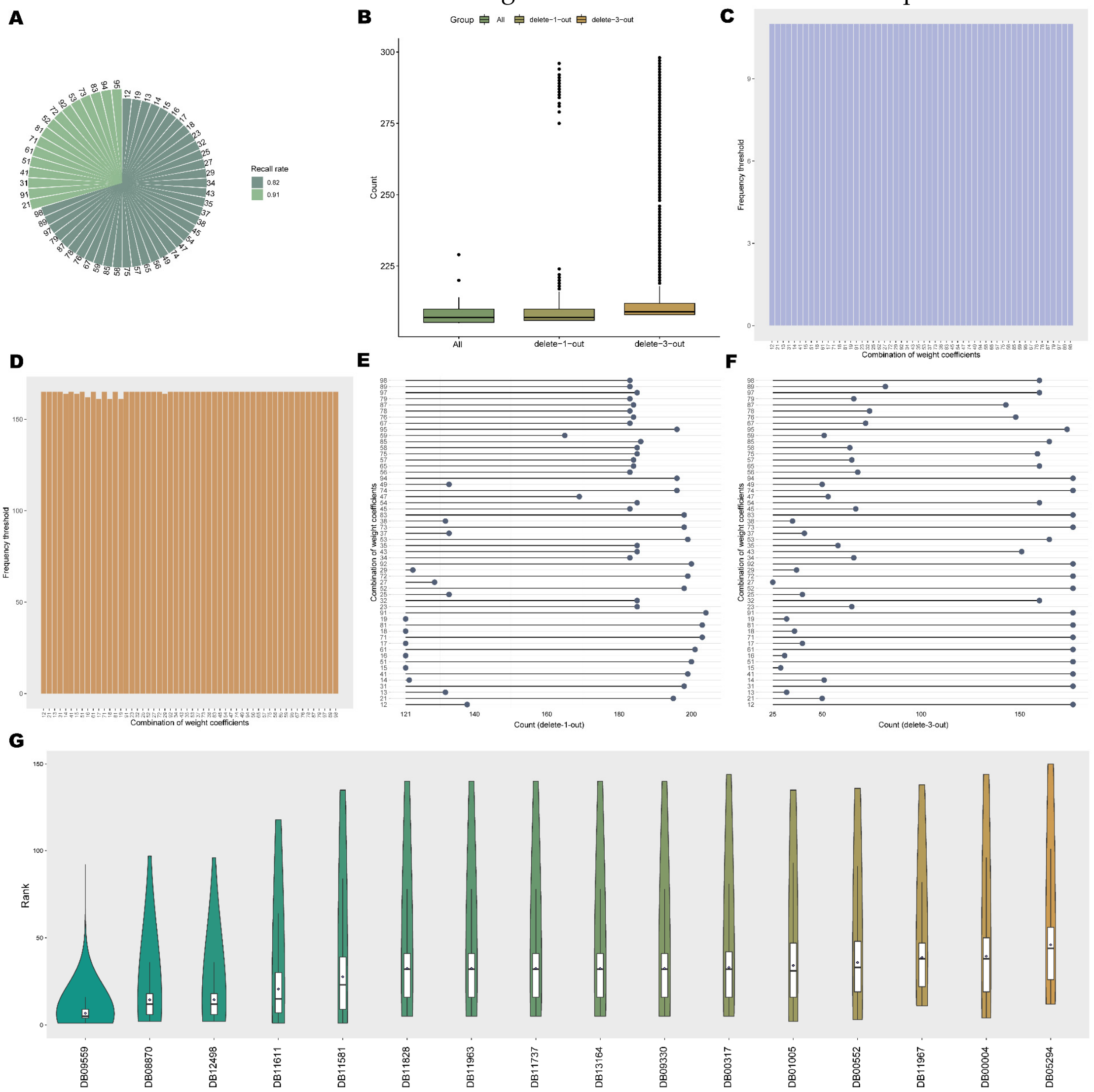

Figure S4

Stable drug candidates in weighted pattern. **(A)** The recall of the leave-one-out cross validation for each weighting pattern. **(B)** Distribution of the number of the top 5% of drugs. **(C-D)** The frequency threshold for

each weighting pattern when the delete-1-out and delete-3-out strategies was implemented. **(E-F)** The number of candidate drugs detected under different weights by the delete-n-out strategy. **(G)** Ranking distribution of stable drug candidates in all weighted patterns. Where the average rank is identified by a purple diamond.

1.3. Potential repositionable drugs for colorectal cancer

By analyzing the stable drug candidates under the unweighted and multiple weighting patterns, 35 and 16 drugs were screened, respectively. The 11 shared drugs were treated as initial predicted drugs (Figure S5A).

Genes *TNFRSF8*, *RRM1*, *EGFR*, and *CCR4* were essential for the survival of colorectal cancer cell lines (Figure S5B). Of these, EGFR was targeted by 6 drugs and 3 essential genes were targeted by 1 drug (Figure S5C). Ultimately, 9 potential repurposable drugs for colorectal cancer targeting 4 druggable targets were predicted. Overall, 9 potential repurposed drugs ranked top in all conditions (Figure S5D). The average rankings were all less than 50.

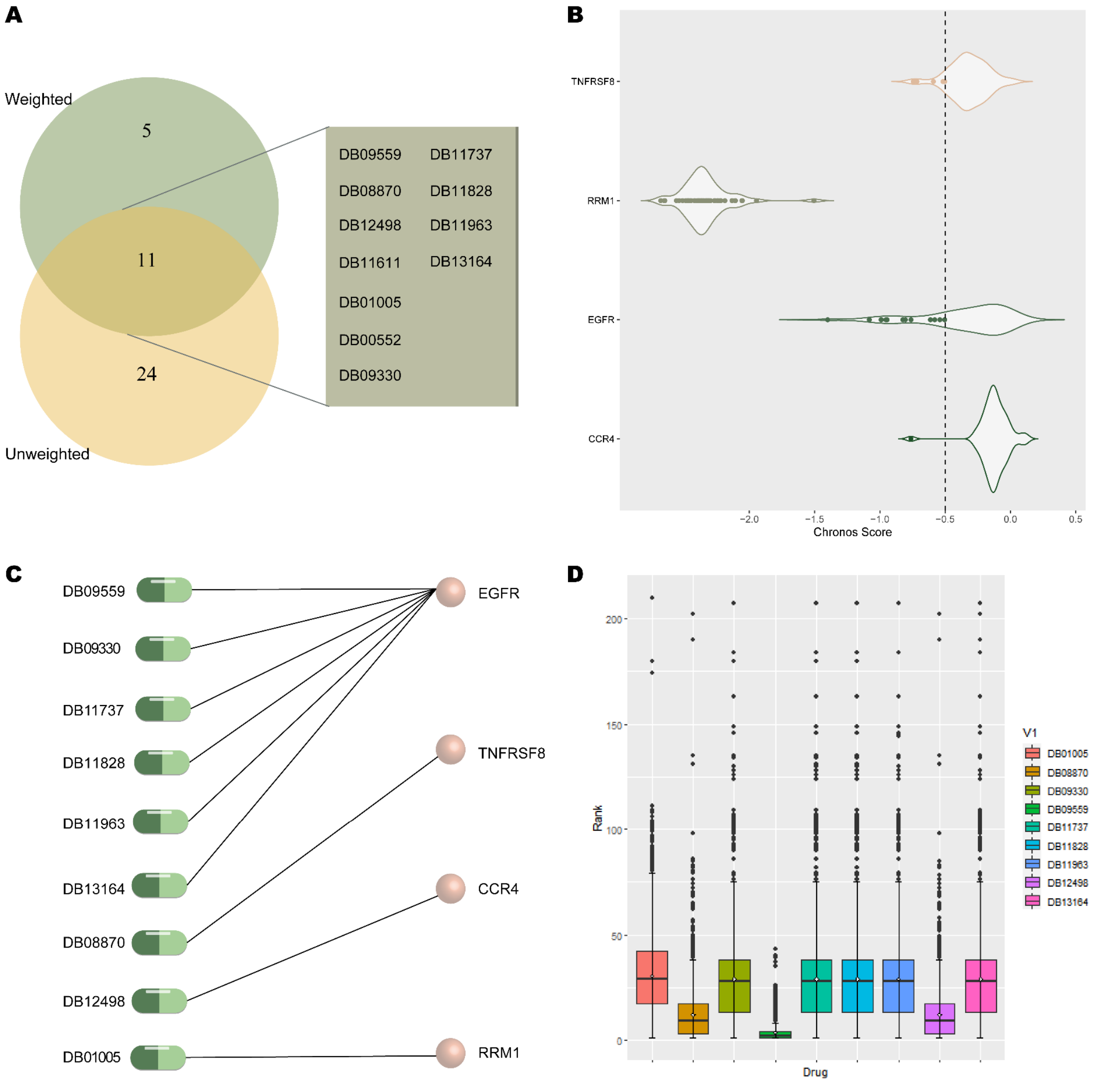

Figure S5

Potential repositionable drugs for colorectal cancer. **(A)** The overlap of

stable drug candidates captured by weighted and unweighted patterns. **(B)** Distribution of essential gene effect scores in colorectal cancer cell lines. Cell lines with gene effect < -0.5 are indicated by highlighted dots. **(C)** Drugs targeting essential genes. **(D)** Ranked distribution of potential repurposed drugs across all conditions.

Of the nine potentially repositionable drugs, seven had corresponding literature support (Table S4).

Table S4

The potential repurposed drugs.

Average Rank	Accession Number	Drug Name	Evidence
3	DB09559	Necitumumab	PMID: 21154125,PMID: 26766738
12	DB08870	Brentuximab vedotin	PMID: 30993587
12	DB12498	Mogamulizumab	PMID:31801624,PMID: 34916725,PMID: 31455681
29	DB09330	Osimertinib	PMID: 31409796
29	DB11737	Icotinib	PMID: 25572529,PMID: 35250582
29	DB11828	Neratinib	PMID: 26243863,PMID: 30203445
29	DB11963	Dacomitinib	PMID: 27733479,PMID: 22249430
29	DB13164	Olmutinib	Unconfirmed
30	DB01005	Hydroxyurea	Unconfirmed

Survival analysis showed that the targets of the 6 predicted drugs were significantly associated with the prognosis of colorectal cancer (Figure S6). The targets of a total of 6 predicted drugs and known therapeutics were mainly enriched in 3 categories of pathways, including colorectal cancer pathway (hsa05210) (Supplementary Figure S7A), tumor progression-related pathways (Supplementary Figure S7B) such as HIF-1 signaling pathway (hsa04066), Choline metabolism in cancer (hsa05231), and Rap1 signaling pathway (hsa04015), MAPK signaling pathway (hsa04010) and other cellular process-related pathways (Supplementary Figure S7C).

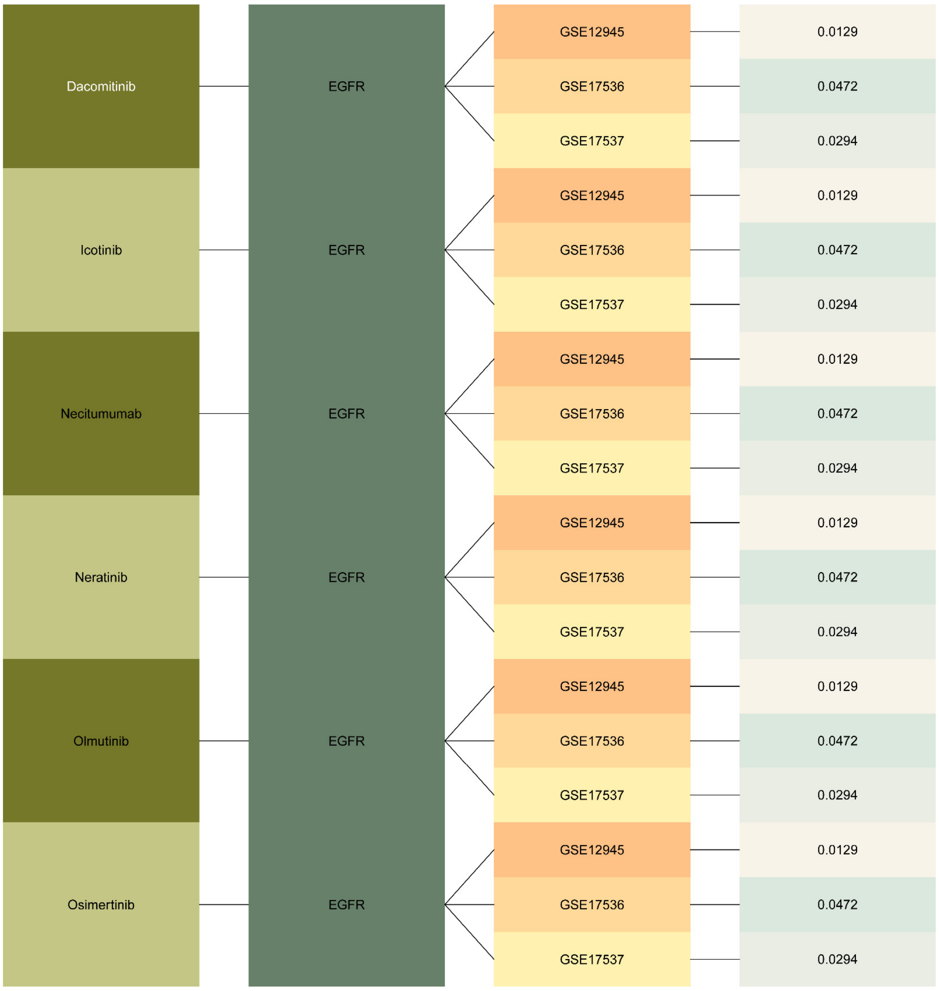

Figure S6

Survival analysis of potential repurposed drugs.

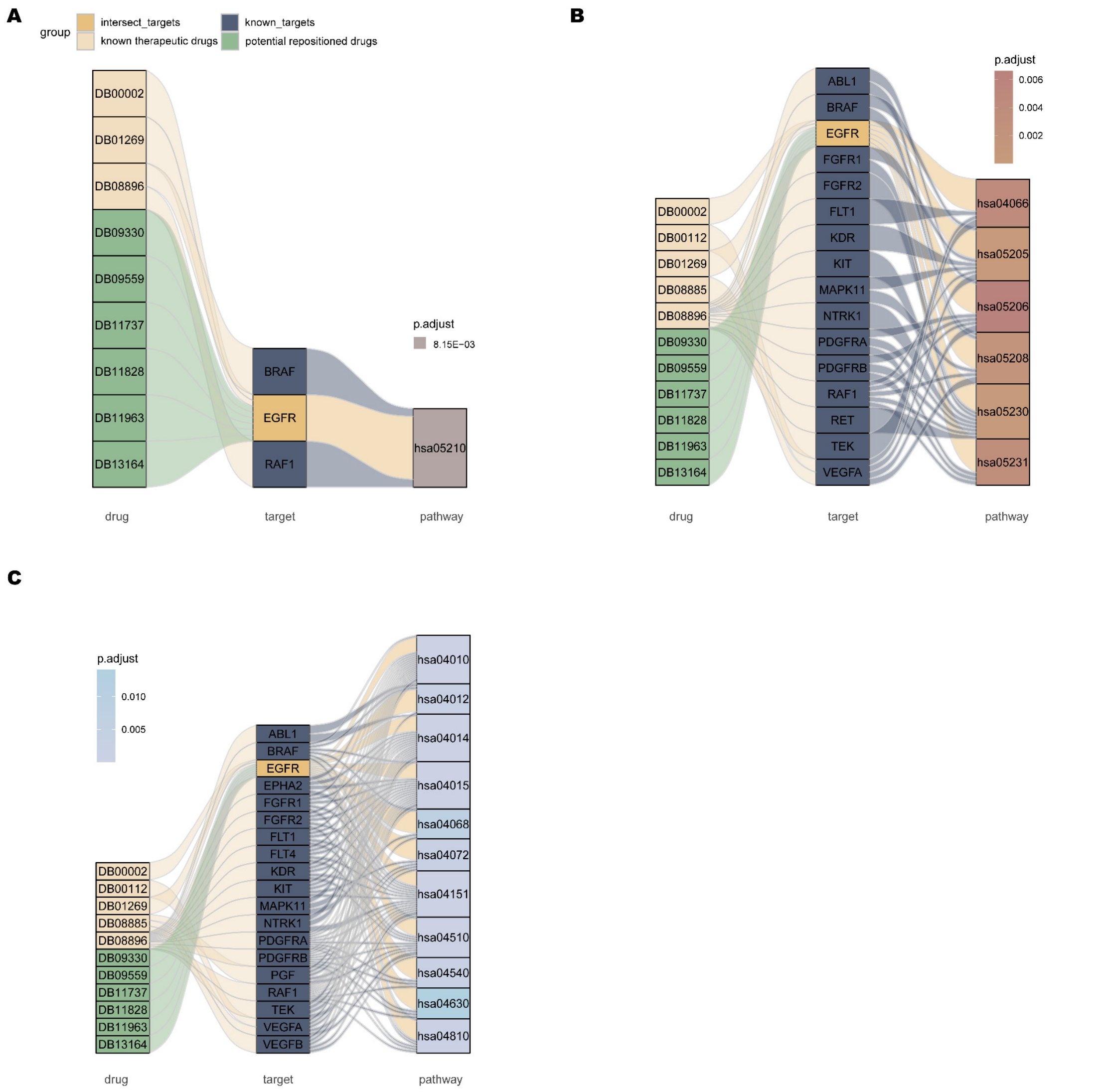

Figure S7

KEGG enrichment analysis. **(A)** colorectal cancer pathway. **(B)** Cancer progression-related pathway. **(C)** Cell process-related pathway.

Databases and software used for this study

1. Network construction part

All code for this work was done in R. To calculate the functional similarity between drugs, the targets of the drugs were retrieved from the DrugBank database[1] and the hallmarks of cancer were retrieved from the MSigdb database[2]. The functional similarity between drugs was calculated by a script written. To calculate the clinical therapeutic similarity between drugs, the xml file of the DrugBank database was downloaded, and the ATC code information of the drugs was extracted using the R package "XML". The clinical therapeutic similarity between drugs was computed by the script written.

2. Identification of potentially repositionable drug part

The xml files of the DrugBank database were downloaded and the indications of the drugs were extracted using the R package "XML". Known therapeutic drugs for non-small cell lung cancer and colorectal cancer were identified by manual review. Scripts were written to enable the calculation of drug-cancer correlation scores and the screening of stable drug candidates. CRISPR gene effect scores and cell line annotation information were obtained from the DepMap database when reviewing the druggable potential of drug candidates.

3. Confirmation of potentially repositionable drugs

Our predictive drugs were confirmed by searching PubMed for published clinical trial articles and information on drug indications recorded in the DrugBank database. The association of predictive drugs with cancer prognosis was examined by two online databases, KM Plotter[3] and PrognoScan[4]. Functional enrichment analysis was implemented using the R package "clusterProfiler"[5] to examine the association between predictive drugs and cancer.

1. Law, V., et al., *DrugBank 4.0: shedding new light on drug metabolism.* Nucleic Acids Res, 2014. **42**(Database issue): p. D1091-7.
2. Liberzon, A., et al., *The Molecular Signatures Database (MSigDB) hallmark gene set collection.* Cell Syst, 2015. **1**(6): p. 417-425.
3. Gyorffy, B., et al., *Online survival analysis software to assess the prognostic value of biomarkers using transcriptomic data in non-small-cell lung cancer.* PLoS One, 2013. **8**(12): p. e82241.
4. Mizuno, H., et al., *PrognoScan: a new database for meta-analysis of the prognostic value of genes.* BMC Med Genomics, 2009. **2**: p. 18.
5. Yu, G., et al., *clusterProfiler: an R package for comparing biological themes among gene clusters.* OMICS, 2012. **16**(5): p. 284-7.
